# AM1172 (a hydrolysis-resistant endocannabinoid analog that inhibits anandamide cellular uptake) reduces the viability of the various melanoma cells, but it exerts significant cytotoxic effects on healthy cells: an in vitro study based on isobolographic analysis

**DOI:** 10.1007/s43440-023-00557-2

**Published:** 2023-11-29

**Authors:** Paweł Marzęda, Paula Wróblewska-Łuczka, Magdalena Florek-Łuszczki, Agnieszka Góralczyk, Jarogniew J. Łuszczki

**Affiliations:** 1 Department of Occupational Medicine, Medical University of Lublin, 20-090 Lublin, Poland; 2 Department of Medical Anthropology, Institute of Rural Health, 20-950 Lublin, Poland

**Keywords:** Cannabinoids, Melanoma, AM1172, Drug interactions, Isobolography, In vitro

## Abstract

**Background:**

Despite great advances in our understanding of the impact of cannabinoids on human organism, many of their properties still remain undetermined, including their potential antineoplastic effects. This study was designed to assess the anti-proliferative and cytotoxic effects of AM1172 (a hydrolysis-resistant endocannabinoid analog that inhibits anandamide cellular uptake) administered alone and in combinations with docetaxel (DOCX), paclitaxel (PACX), mitoxantrone (MTX) and cisplatin (CDDP) on various human malignant melanoma A375, FM55P, SK-MEL 28 and FM55M2 cell lines.

**Materials:**

In the MTT, LDH, and BrdU assays, the potency and safety of AM1172 when administered alone and in combinations with DOCX, PACX, MTX, and CDDP were determined.

**Results:**

The isobolographic analysis revealed that combinations of AM1172 with PACX, DOCX, MTX, and CDDP exerted additive interactions, except for a combination of AM1172 with PACX in primary melanoma A375 cell line, for which synergy was observed (*p<0.05). Nevertheless, AM1172 when administered alone produced cytotoxic effects on healthy human melanocytes (HEMa-LP) and human keratinocytes (HaCaT), which unfortunately limits its potential therapeutic utility.

**Conclusions:**

AM1172 cannot be used separately as a chemotherapeutic drug, but it can be combined with PACX, DOCX, MTX, and CDDP, offering additive interactions in terms of the anti-proliferative effects in various malignant melanoma cell lines.

**Supplementary Information:**

The online version contains supplementary material available at 10.1007/s43440-023-00557-2.

## Introduction

Melanoma is the most aggressive skin cancer whose incidence still tends to increase [1, 2]. In the early stages, melanoma can be effectively treated surgically, with a 5-year survival rate up to 99%. However, the survival rates drop drastically to about 30% when distant metastases occur [3, 4]. Melanoma harbors one of the highest mutation frequencies among human cancers, it has high invasiveness and is likely to give metastases to the brain [5, 6].

In recent years the treatment outcomes of patients with melanoma have greatly improved, mostly due to the invention of immunotherapy, which involves anti-PD-1 agents, anti-cytotoxic T lymphocyte antigen-4 (anti-CTLA-4) antibodies, and a combination of BRAF/MEK targeted therapy [7–10]. However, some patients suffer from drug-induced toxicity or from hyperprogression of the disease, which although is a rare phenomenon, it needs aggressive treatment and special clinical attention [10–13]. The toxicity of chemotherapeutic drugs along with the high cost of immunotherapy with novel drugs, prompted researchers and clinicians to search for novel treatment alternatives, based on natural products [14].

Overwhelming evidence indicates that plant-derived cannabinoids from *Cannabis sativa* spp. (phytocannabinoids) have a solid and firmed position in traditional medicine in the treatment of cancers [15]. Cannabinoids bind to several receptors in mammalian organisms, including cannabinoid CB1 and CB2 receptors, peroxisome proliferator-activated receptor (PPAR)α and PPARγ receptors, transient receptor potential vanilloid-1 (TRPV1) channels and G-protein coupled receptor (GPR)55 and GPR35 receptors [16, 17].

Numbers of experimental studies indicate some favorable effects of various (naturally-occurring and synthetic) cannabinoids used in the treatment of melanoma. For instance, a naturally-occurring Δ^9^THC (delta-^9^-tetrahydrocannabinol) and synthetic WIN 55,212-2 (a full agonist at the CB_1_ receptor, PPARα and PPARγ receptors) decreased the viability of melanoma B16 and A375 cell lines [18]. Additionally, WIN 55,212-2 exerted anti-proliferative effects on the metastatic melanoma SK-MEL 28 cell line and the ocular uveal melanoma OCM-1 cell line [19]. In another study, cannabidiol (CBD – a naturally-occurring non-psychoactive cannabinoid) considerably reduced the cell growth of B16 melanoma cells [20]. Both, CBD and Δ^9^THC (when combined at the fixed-ratio of 1:1) reduced cell viability, in a concentration-dependent manner, in different various primary and metastatic melanoma cell lines (A375, SBcl2, SK-MEL 28, UACC-62, Colo-800 and A2058) [21]. CBD administered alone suppressed cell proliferation in various human malignant melanoma cell lines (A375, FM55P, SK-MEL 28 and FM55M2) [22]. In in vivo studies, monotherapy with Δ^9^THC significantly inhibited tumor growth of transplanted HCmel12 melanoma, but not that in B16 melanoma in mice [23]. Additionally, it has been found that CBD monotherapy prolonged survival in mice with B10F10 melanoma tumors [24]. In a xenograft mouse model, both CBD and Δ^9^THC (in a fixed ratio of 1:1) substantially inhibited viability, proliferation, and tumor growth in CHL-1 melanoma cells [25]. Similarly, arvanil and olvanil (two synthetic ligands for TRPV1 and CB1 receptors) reduced cell viability in different human malignant melanoma cell lines (A375, SK-MEL 28, FM55P, and FM55M2) [26]. Additionally, it has been reported that *N*-arachidonoyl-ethanolamine, also known as anandamide (AEA – an endogenous cannabinoid) produced cytotoxicity in A375 melanoma cells, which was enhanced by the inhibition of fatty acid amide hydrolase (FAAH), an enzyme degrading AEA [27]. Of note, AEA is the CB1 and CB2 receptor agonist, putative GPR55 agonist, moreover it binds to L-type Ca2+ channels, PPARα and PPARγ receptors, and it was the first identified endogenous TRPV1 agonist [28, 29]. Similarly, palmitoylethanolamide (PEA – an endocannabinoid-like lipid mediator) reduced the viability of B16 mouse melanoma cells, which was enhanced by the inhibition of FAAH and thus, the inhibition of PEA hydrolysis [30]. On the other hand, URB447 – a synthetic cannabinoid ligand acting as a CB2 agonist and CB1 antagonist reduced cell viability of melanoma leading to G0/G1 cell cycle arrest accompanied by a decrease in S-phase cell counts [31]. Generally, the activation of CB2 receptors on melanoma cells can lead to G1-cell cycle arrest by inhibiting Akt/pRb signaling molecules, activation of caspase-3, and stimulation of ROS production [32]. Of note, the CB2 receptors in melanoma cells are overexpressed [33].

**Fig. 1:**
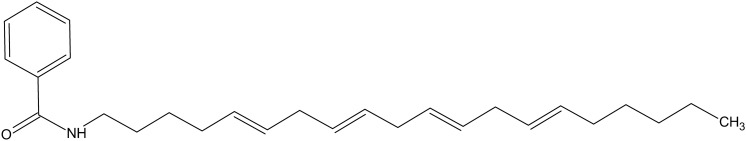
Chemical structure of AM1172

Considering these facts, little is known about the possibility of modifying the endocannabinoid system in mammalian organisms to enhance the anticancer properties of endocannabinoids. One of the tool substances affecting the endocannabinoid system in mammalian organisms is AM1172 (N-5Z,8Z,11Z,14Z-eicosatetraenyl-4-hydroxy-benzamide; Fig. 1).

AM1172 is the metabolically stable inhibitor of AEA transport, FAAH inhibitor, and fatty acid binding proteins (FABPs) inhibitor [34–36]. AM1172 elevates AEA levels through different mechanisms, which potentially leads to the enhancement of the AEA-induced cytostatic effects, which in the setting of melanoma treatment are reduction of melanoma cells viability by induction of apoptosis, G2/M cell cycle arrest, and cell necrosis [27, 37, 38]. AM1172 elevates endocannabinoid concentrations, and, therefore, the drug produces cannabinoid-like effects and enhances the effects of exogenously administered cannabinoids [39]. Additionally, the inhibition of enzymes involved in the degradation of endocannabinoids (i.e., FAAH) is expected to enhance the effects evoked by endocannabinoids due to the increased concentration of endocannabinoids in the sites of their action(s) and/or prolongation of the action of endocannabinoids as a result of a long-term availability of endocannabinoids, which through the blockade of cellular uptake can bind specifically to the CB1, CB2, PPARα, PPARγ, TRPV1, GPR55, and GPR35 receptors.

In experimental and clinical oncology, the main rule when choosing anticancer drugs for chemotherapy protocols and treatment regimens is to select the drugs with various molecular mechanisms of action that the drugs could be able to destroy as many cancerous cells as possible [40, 41]. Because the molecular mechanisms of action of AM1172 considerably differ from the molecular mechanisms of other tested chemotherapeutics, such a “gold rule” inspired us to combine some chemotherapeutics (including, cisplatin (CDDP), mitoxantrone (MTX), docetaxel (DOCX), and paclitaxel (PACX)) with AM1172 (a hydrolysis-resistant endocannabinoid analog that inhibits AEA cellular uptake) to verify whether the two-drug combinations would be efficient in suppressing proliferation of malignant melanoma cells in the in vitro MTT assay.

Previously, it has been documented that CBD, arvanil, and olvanil inhibited cell proliferation in various human malignant melanoma cell lines (A375, FM55P, SK-MEL 28 and FM55M2) [22, 26]. We sought therefore to continue our previous studies by assessing the antiproliferative potential of AM1172 (another compound that affects the endocannabinoid system) to shed more light on the antiproliferative effects of cannabinoid ligands in various malignant melanoma cell lines. AM1172 was chosen as a tool substance for this experiment because it modulates the endocannabinoid system via irreversible blocking of cellular AEA uptake and, through the increase in AEA content, activation of the endocannabinoid system.

This study was designed for two main purposes: (1) to determine whether AM1172 possesses anti-proliferative effects on various malignant melanoma (primary and metastatic) cell lines (A375, SK-MEL 28, FM55P, and FM55M2), being simultaneously safe on normal human melanocytes (HEMa-LP) and human keratinocytes (HaCaT); (2) to establish whether AM1172 can synergistically cooperate with 4 selected chemotherapeutic drugs (MTX, CDDP, DOCX, and PACX) in terms of reduction of cell viability of 4 various melanoma cell lines (A375, SK-MEL 28, FM55P and FM55M2) in the MTT assay. The evaluation of interactions between AM1172 and the tested chemotherapeutic drugs was assessed by the isobolographic analysis, which is the best method used when evaluating drug–drug interactions in cancer studies [42, 43]. The rationale for investigating the joint treatments of AM1172 with DOCX, PACX, CDDP, and MTX was based on theoretical presumptions that drugs with different molecular mechanisms of action concerning cell toxicity should mutually potentiate their cytotoxic effects and significantly inhibit cell proliferation. In clinical conditions, chemotherapeutic regimens and drug treatment protocols are usually based on the joint application of two or three drugs with different mechanisms of action to gain the maximum possible anti-proliferative effect [44–46]. Although the modern chemotherapy for malignant melanoma is based on carboplatin, temozolomide, DOCX, PACX, and various humanized monoclonal antibodies (INN-mabs), we choose some classic chemotherapeutics (i.e., CDDP, DOCX, PACX, and MTX) to test with AM1172, and to compare the results from this study with those for other cannabinoid ligands.

Of note, in in vitro studies PACX stabilizes and prevents microtubules depolymerization, leading to cell cycle arrest at the G2/M phase and cell death [47]. PACX induces apoptosis by affecting p38 mitogen-activated protein kinase (p38 MAPK), extracellular signal-regulated kinase (ERK), nuclear factor-kappa B (NF-κB) and c-Jun N-terminal kinase or stress-activated protein kinase (JNK/ SAPK) pathways [48]. DOCX inhibits not only microtubular depolymerization, but also attenuates the effects of bcl-2 and bcl-xL gene expression [49]. MTX (as a type II topoisomerase inhibitor) disrupts DNA synthesis and DNA repair by intercalating between DNA bases [50]. CDDP binds in DNA and forms intra-strand DNA adducts leading to the inhibition of DNA synthesis and cell growth [51]. CDDP-induced DNA damage contributes to cell apoptosis by activating oxidative stress and several signaling pathways, including mitogen-activated protein kinase (MAPK), Jun N-terminal kinases (JNK) or Akt pathways [52]. Theoretically, DOCX, PACX, CDDP, and MTX due to their various molecular mechanisms of action should contribute to the enhanced cytotoxicity evoked by the combinations of the chemotherapeutic drugs with AM1172 in malignant melanoma cells.

## Materials and methods

### Cell lines

The SK-MEL 28 (ATCC HTB-72) (metastatic) and A375 (ATCC CRL-1619) (primary) malignant melanoma cell lines were purchased from the American Type Culture Collection (ATCC, Manassas, Virginia, USA) and cultured in the Eagle’s minimal essential medium (EMEM – ATCC 30-2003-500 ml, Lot No. 80420213) and Dulbecco’s Modified Eagle’s Medium—high glucose (DMEM – D6546-500 ml, Lot No. RNBL0124) (both from Sigma-Aldrich, St. Louis, MA, USA), respectively. The FM55P (ECACC 13012417) (primary) and FM55M2 (ECACC 13012559) (metastatic) malignant melanoma cell lines were purchased from the European Collection of Cell Cultures (ECACC, Salisbury, UK)) and cultured in RPMI—1640 Medium (R8758-500 ml, Lot No. RNBK1487) (Sigma-Aldrich, St. Louis, MO, USA). Each culture medium was supplemented with 10% Fetal Bovine Serum (FBS – F9665-500 ml, Lot No. BCBS0536V) (Sigma-Aldrich, St. Louis, MO, USA) and 1% of penicillin/streptomycin (P4333-100 ml) (Sigma-Aldrich, St. Louis, MO, USA). All cultures were kept at 37 °C in a humidified atmosphere of 95% air and 5% CO_2_. The cells grew to 80% confluence.

### Drugs

Paclitaxel (PACX – T1912-25 mg, Lot No. SLBZ3160), mitoxantrone (MTX hydrochloride – M2305000–60 mg), and docetaxel (DOCX – PHR1883-200 mg) (Sigma-Aldrich, St. Louis, MO, USA) were dissolved in DMSO (D2650-100 ml, Lot No. RNBH0690) as stock solutions. Cisplatin (CDDP – PHR1624-200 mg, Lot No. LRAB7778) (Sigma-Aldrich, St. Louis, MO, USA) was dissolved in phosphate-buffered saline (PBS – 806552-500 ml, Lot No. RNBH7571) with Ca^2+^ and Mg^2+^. AM1172 (Cat. No. 3381-10 mg) (Tocris, Bristol, UK) was dissolved in ethanol as a stock solution at a concentration of 5 mg/mL. The drugs were dissolved to the respective concentrations with a culture medium before the use.

### Cell viability assessment

The HaCaT cell line – normal human keratinocyte cells (density: 1 × 10^4^ cells/mL), HEMa-LP cell line – normal human melanocytes (density: 5 × 10^3^ cells/mL) and four human malignant melanoma lines (A375, SK-MEL 28, FM55M2, FM55P) (density: 2–3 × 10^4^ cells/mL, depending on the cell line) were plated on microtiter plates (NEST Biotechnology, Wuxi, China) The next day, the culture medium was removed and cells were exposed to serial dilutions of PACX, DOCX, AM1172, CDDP and MTX in a fresh culture medium. Cell viability was assessed after 72 h using the MTT test, as described earlier [26]. Each treatment was performed in triplicate and each experiment was repeated 3 times to ensure repeatability and validity of the results. Of note, the 72 h incubation time is the average doubling time for all melanoma cell lines tested. In the case of the A375 cell line, this time is the shortest 6–12 h [53], for the SK-MEL 28 cell line it is 17.5 h [54], but the FM55M2 and FM55P cell lines are an incubation time of 72 h [22, 55, 56].

### Cytotoxicity assessment–LDH assay

Optimized amounts of SK-MEL 28 (3 × 10^4^/mL), A375 (2 × 10^4^/mL), FM55P (2 × 10^4^/mL), FM55M2 (2 × 10^4^/mL), normal human keratinocytes HaCaT (1 × 10^4^/mL) and normal human melanocytes HEMa-LP (density: 5 × 10^3^ cells/mL) cells were placed on 96-well plates (Nunc, Roskilde, Denmark). After a day cells were washed and then exposed to increasing concentrations of AM1172 in the proper fresh culture medium for 72 hours of exposure. Data about the cytotoxicity was obtained by measuring cytoplasmic lactate dehydrogenase (LDH) activity released from the damaged cells after exposure. LDH assay was performed according to the manufacturer’s instruction (Cytotoxicity Detection KitPLUS LDH) (Roche Diagnostics, Mannheim, Germany), as described earlier [22, 26, 56].

### Cell proliferation assay

Cell Proliferation Elisa, BrdU Kit (Roche Diagnostics, Mannheim, Germany) was performed by following the manufacturer’s instructions. Optimized amounts of A375 (2 × 10^4^/mL) SK-MEL 28 (3 × 10^4^/mL), FM55P (2 × 10^4^/mL), FM55M2 (2 × 10^4^/mL), and normal human keratinocytes HaCaT (1 × 10^4^/mL) cells were placed on a 96-well plate (Nunc, Roskilde, Denmark). The next day, the melanoma cells were treated with increased concentrations of AM1172 for 48 h. After this exposure, the 10 µL/well BrdU Labeling Solution (100 µM) was added and cells were reincubated for another 24 h at 37 °C. In the final part, the culture medium was removed and cells were fixed in FixDenat solution (200 µL/well) (30 min, at RT), then the working solution of anti-BrdU antibody coupled with horseradish peroxidase (anti-BrdU-POD) were subsequently added (100 µL/well) (90 min, RT) and detected using tetramethylobenzidine substrate (TMB) (100 µL/well) (30 min, RT). To stop the enzymatic reaction, a total of 1 M (25 µL/well) sulfuric acid was added, as described earlier [22, 26, 56].

### Isobolographic analysis of interactions

Log-probit analysis was used to determine the percentage of inhibition of cell viability (transformed into probit) and concentrations of AM1172 when administered singly in the A375, SK-MEL 28, FM55P, and FM55M2 melanoma cell lines (transformed into logarithm of concentrations), as reported earlier [56]. The next step was based on the calculation of the median inhibitory concentrations (IC_50_ values) of AM1172 from the log-probit concentration–-response lines [57]. A linear model of Loewe’s additivity allowed to verify of the parallelism of concentration–response curves for the studied drugs – AM1172, with PACX, DOCX, CDDP or MTX, as reported earlier [58–61]. Verification of parallelism confirmed that none of the tested two-drug combinations had their lines mutually collateral in each combination in in vitro MTT assay. The type I isobolographic analysis for non-parallel concentration–response effect lines, defines the additivity as an area bounded by two lower and upper isoboles of additivity [59–65]. The median additive inhibitory concentrations (IC_50 add_) for the two-drug mixture of AM1172, with PACX, DOCX, CDDP or MTX, which theoretically should inhibit 50% of cell viability, were calculated as demonstrated earlier [58, 61]. The final step in isobolography was based on the determination of the experimentally derived IC_50mix_ (at the fixed ratio of 1:1) in malignant melanoma cell lines measured in vitro in the MTT assay.

### Statistical analysis

The statistical analysis of data was performed with the GraphPad Prism 8.0 Statistic Software (San Diego, CA, USA). Results from the MTT, LDH, and BrdU assays were analyzed with a one-way ANOVA test followed by Dunnett’s significance test. The data are expressed as means ± standard error (SEM) and statistical significance was indicated with asterisks (*p<0.05, **p<0.01, ***p<0.001, and ****p<0.0001). The experimentally derived IC_50mix_ values for the mixture of AM1172 with PACX, DOCX, MTX, or CDDP were statistically compared with their respective theoretically additive IC_50add_ values using the unpaired Student’s t-test with Welch’s correction, as described elsewhere [66, 67].

## Results

### Influence of AM1172 on cell viability in the MTT test

**Fig. 2:**
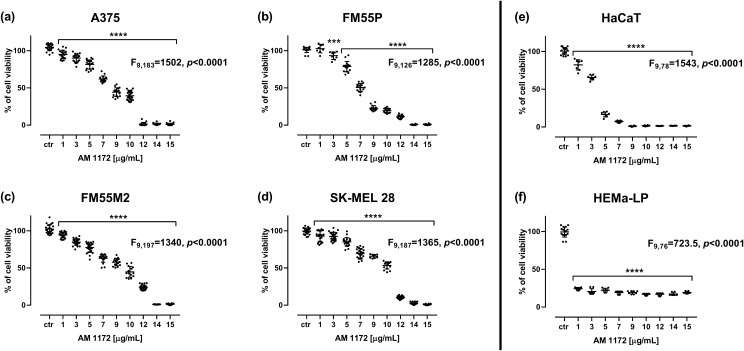
The impact of AM1172 on the viability of malignant melanoma cell lines [A375 (**A**), SK-MEL 28 (**B**), FM55P (**C**) and FM55M2 (**D**)], normal human keratinocytes (HaCaT) (**E**), and normal human melanocytes (HEMa-LP) (**F**), measured by means of the MTT assay after 72 h. Bars represent mean ± SEM (***p<0.001 and ****p<0.0001). Each experiment was repeated 3 times to ensure repeatability and validity of the results. Statistical analysis was performed with a one-way ANOVA test followed by Dunnett’s post-hoc test.

Incubation of various human melanoma cell lines (i.e., A375, SK-MEL 28, FM55P, and FM55M2) with AM1172 resulted in a reduction of the viability of cells in a concentration-dependent manner (Fig. 2A–F). None of the solvents used in the respective control groups (i.e. phosphate-buffered saline (PBS), dimethyl sulfoxide (DMSO), and ethanol), applied in relevant concentrations did not affect the viability of melanoma cells (data not shown). The experimentally derived median inhibitory concentration (IC_50_) values for AM1172 in various melanoma cell lines are as follows: for the A375 cell line – 20.41 ± 1.63 µM, for the FM55P cell line – 16.88 ± 0.98 µM, for the FM55M2 cell line – 21.44 ± 1.64 µM, and for the SK-MEL 28 cell line – 22.85 ± 1.74 µM. The IC_50_ value for AM1172 in the HaCaT cell line in the MTT was 2.76 ± 0.91 µM, and the IC_50_ value for AM1172 in the HEMa-LP cell line in the MTT was 0.49 ± 0.24 µM. Of note, the experimentally derived IC_50_ values for DOCX, PACX, CDDP, and MTX in four various malignant melanoma cell lines have been determined in our earlier studies [22, 56, 68].

### Cytotoxicity of AM1172 in the LDH test

**Fig. 3:**
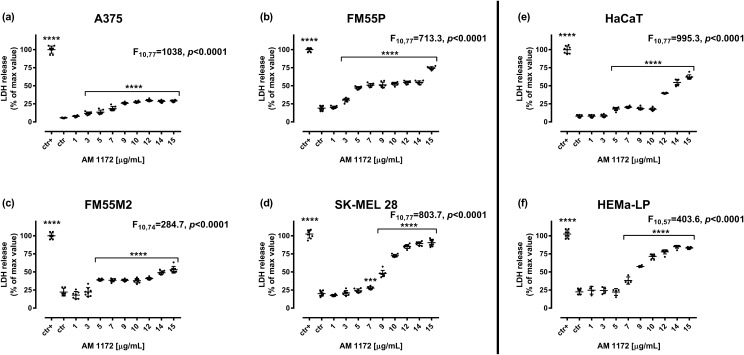
Cytotoxicity of AM1172 to malignant melanoma cells: A375 (**A**), SK-MEL 28 (**B**), FM55P (**C**) and FM55M2 (**D**), normal human keratinocytes HaCaT (**E**), and normal human melanocytes HEMa-LP (**F**). Lactate dehydrogenase ELISA kit was used to quantify cytotoxicity by measuring LDH activity released from damaged cells. Normal keratinocytes, melanocyte cells and malignant melanoma cells were incubated for 72 h alone or in the presence of AM1172 (1-15 µg/mL). The results are presented as the percentage in LDH release to the medium by treated cells versus cells grown in control medium (ctr) and cells treated with Lysis buffer (ctr+). Bars represent mean ± SEM (***p<0.001 and ****p<0.0001). Each experiment was repeated 3 times to ensure repeatability and validity of the results. Statistical analysis was performed with a one-way ANOVA test followed by Dunnett’s post-hoc test.

Cytotoxicity of AM1172 to normal human keratinocytes (HaCaT), normal human melanocytes (HEMa-LP), and malignant melanoma cells (A375, SK-MEL 28, FM55P, and FM55M2) was quantified by LDH assay. This test allowed us to assess and measure the release of lactate dehydrogenase into the medium after a certain time of incubation. The LDH levels indicated damage to the cell membrane and cell death [69]. In our experiment, the diagrams show that the cytotoxicity of AM1172 in various malignant melanoma cell lines grows, in a concentration-dependent manner, with a significant leakage in the SK-MEL 28, FM55P, and FM55M2 cell lines, where the impact on SK-MEL 28 cell line was the greatest (Fig. 3A–D). Moreover, the significant cytotoxicity of AM1172 was observed in healthy human cells: the HEMa-LP and HaCaT cell lines (Fig. 3E, F).

### Influence of AM1172 on cell proliferation in the BrdU test

**Fig. 4:**
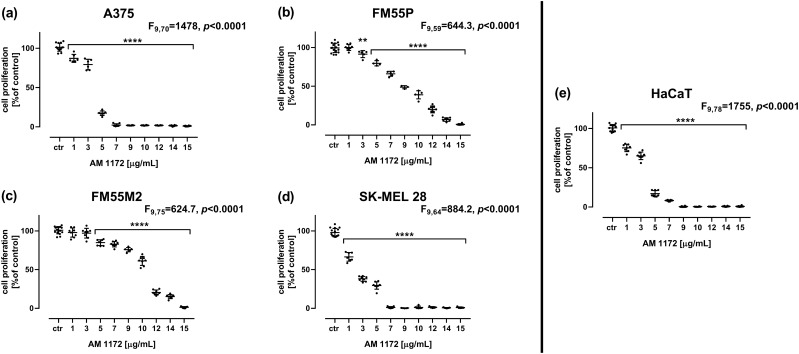
The effect of AM1172 on the proliferation of malignant melanoma cell lines [A375 (**A**), SK-MEL 28 (**B**), FM55P (**C**), FM55M2 (**D**)], and normal human keratinocytes (HaCaT) (**E**) measured by means of BrdU assay after 72 h. Bars represent mean ± SEM (**p<0.01, ****p<0.0001). Each experiment was repeated 3 times to ensure repeatability and validity of the results. Statistical analysis was performed with a one-way ANOVA test followed by Dunnett’s post-hoc test.

In the BrdU assay, AM1172 inhibited the proliferation of all tested malignant melanoma cell lines (A375, SK-MEL 28, FM55P, and FM55M2 – Fig. 4A–D) and normal human keratinocytes HaCaT (Fig. 4E). However, the inhibition of proliferation was significantly lower in the FM55P and FM55M2 cell lines than in normal human keratinocytes HaCaT, and thus higher concentrations of AM1172 (3-times higher for the line FM55P and 5-times higher for the line FM55M2) were needed to obtain the similar effect (Fig. 4A–E).

### Interactions of AM1172 with chemotherapeutic drugs in the MTT test

The characteristics of interactions for the mixtures of AM1172 with one of the tested chemotherapeutics (DOCX, PACX, CDDP, or MTX) administered at the fixed-ratio of 1:1 was examined in the MTT assay in four melanoma cell lines (A375, SK-MEL 28, FM55P and FM55M2). All the mentioned cell lines were incubated with proportionally increasing concentrations of AM1172 and DOCX, PACX, CDDP, or MTX. The log-probit analysis of concentration-response anti-proliferative effects produced by the respective two-drug mixtures allowed for calculating the experimentally derived IC_50mix_ values for the combinations studied in the MTT assay. The obtained results presented the concentration-dependent reduction in malignant melanoma cell viability (Supplementary Figures 1-4). The test of parallelism between the concentration-response lines for the studied drugs (AM1172+DOCX, AM1172+PACX, AM1172+CDDP, and AM1172+MTX) revealed that all the concentration-response lines are not collateral to each other in all the studied melanoma cell lines (Supplementary Figures 1-4).

**Fig. 5:**
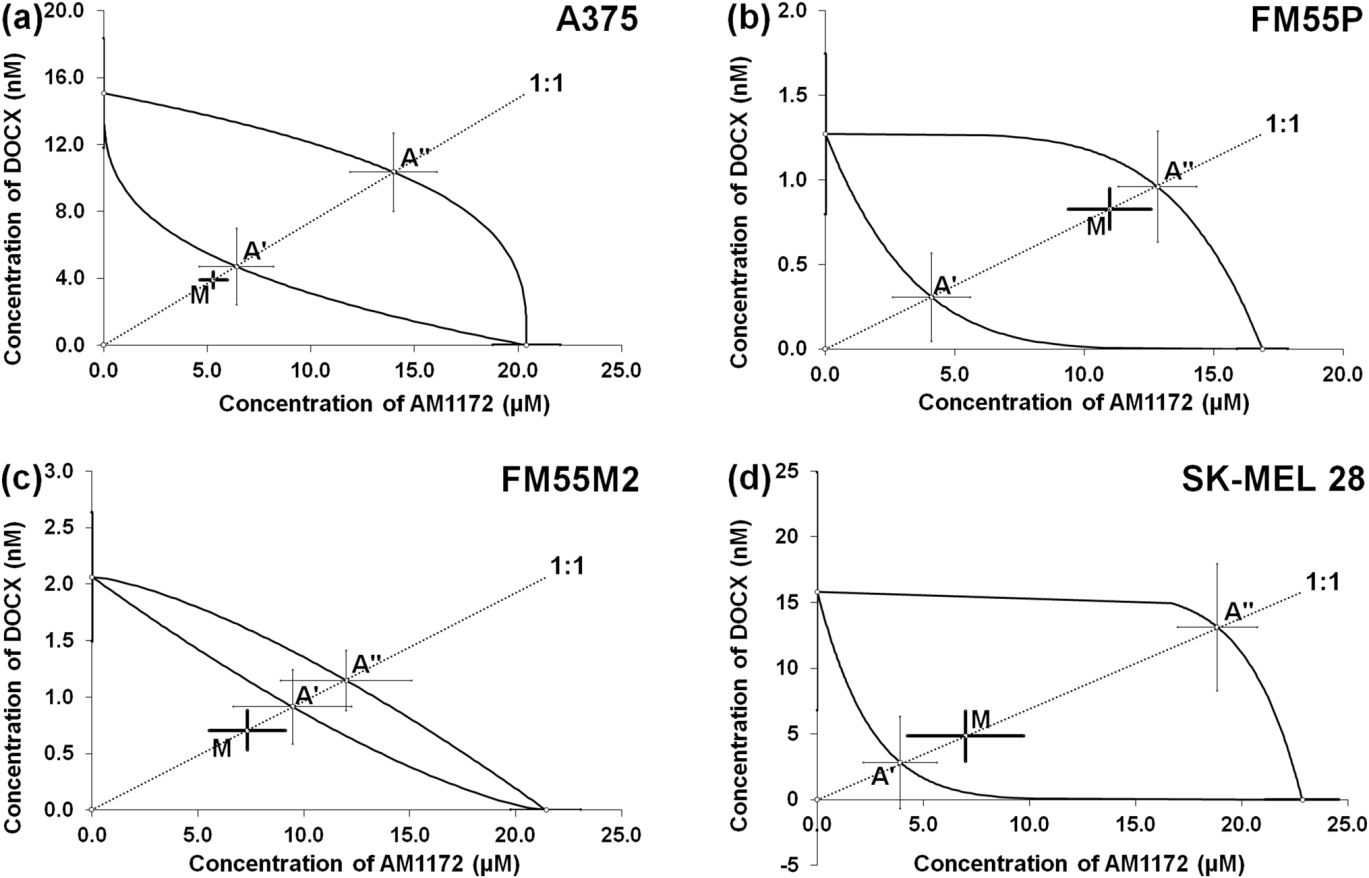
Isobolograms showing interactions between AM1172 and docetaxel (DOCX) concerning their anti-proliferative effects on A375 (**a**), FM55P (**b**), FM55M2 (**c**), and SK-MEL 28 (**d**) malignant melanoma cell lines measured in vitro by the MTT assay. Points A’ and A” depict the theoretically calculated IC_50add_ values for both lower and upper isoboles of additivity, respectively. Point M represents the experimentally derived IC_50mix_ values for the total concentration of the mixture of AM1172 with DOCX that produced a 50% anti-proliferative effect in malignant melanoma cell lines measured *in vitro* by the MTT assay. Statistical analysis was performed with Student’s t-test followed by Welch’s correction.

The isobolographic analysis of interaction for non-parallel concentration-response lines revealed that the combination of AM1172 with DOCX at the fixed ratio of 1:1 exerted additive interactions in all the tested malignant melanoma (A375, FM55P, SK-MEL 28, and FM55M2) cell lines (Table 1, Fig. 5A–D).

**Table 1:** Isobolographic analysis of interactions between AM1172 and DOXC, PACX, CDDP, and MTX (at the fixed ratio of 1:1) for nonparallel concentration–response effects in various malignant melanoma cell lines.

Drug combination	Cell line	IC_50mix_ [µM]	n_mix_	Lower IC_50add_ [µM]	n_add_	Upper IC_50add_ [µM]	Student’s t-test Welch’s correction	Interaction
AM1172 + DOCX	FM55P	11.81 ± 1.71	72	4.40 ± 1.76	188	13.78 ± 1.84	t_219_=0.786, *p*=0.433	Additive
	FM55M2	8.04 ± 1.97	48	10.38 ± 3.11	188	13.14 ± 3.25	t_224_=0.635, *p*=0.526	Additive
	A375	9.22 ± 1.14	72	11.12 ± 4.06	188	24.35 ± 4.42	t_214_=0.450, *p*=0.653	Additive
	SK-MEL 28	11.82 ± 4.62	72	6.74 ± 5.24	188	31.99 ± 6.70	t_228_=0.729, *p*=0.467	Additive
AM1172 + PACX	FM55P	11.09 ± 0.77	72	10.49 ± 1.56	188	11.00 ± 1.60	t_249_=0.052, *p*=0.958	Additive
	FM55M2	7.79 ± 1.22	48	13.54 ± 3.44	188	14.24 ± 3.49	t_223_=1.573, *p*=0.117	Additive
	A375	5.15 ± 0.81 *	72	9.31 ± 1.68	188	11.22 ± 1.85	t_249_=2.229, *p*=0.027	Synergistic
	SK-MEL 28	11.11 ± 2.62	72	10.48 ± 6.25	188	30.46 ± 6.67	t_239_=0.093, *p*=0.926	Additive
AM1172 + CDDP	FM55P	15.00 ± 1.55	72	4.00 ± 1.31	164	14.34 ± 1.49	t_191_=0.305, *p*=0.761	Additive
	FM55M2	15.43 ± 1.54	72	7.39 ± 2.05	164	15.82 ± 2.33	t_234_=0.140, *p*=0.889	Additive
	A375	17.43 ± 3.27	72	5.83 ± 2.11	188	15.88 ± 2.19	t_138_=0.394, *p*=0.694	Additive
	SK-MEL 28	14.36 ± 2.00	72	11.63 ± 3.14	164	14.55 ± 3.29	t_233_=0.048, *p*=0.962	Additive
AM1172 + MTX	FM55P	8.01 ± 1.37	72	5.28 ± 1.64	188	11.94 ± 1.90	t_253_=1.678, *p*=0.095	Additive
	FM55M2	6.47 ± 1.24	72	9.64 ± 1.90	188	11.98 ± 2.01	t_257_=1.397, *p*=0.164	Additive
	A375	19.85 ± 2.40	72	4.60 ± 2.71	188	15.89 ± 3.04	t_244_=1.022, *p*=0.308	Additive
	SK-MEL 28	6.40 ± 3.05	72	6.01 ± 2.48	188	18.58 ± 2.68	t_168_=0.098, *p*=0.922	Additive

The IC_50_ values (in µM ± SEM) for the mixture of AM1172 with DOCX, PACX, CDDP, and MTX were determined experimentally (IC_50mix_) in four melanoma malignant cell lines in the in vitro MTT assay. The IC_50add_ values were calculated from the lower and upper isoboles of additivity. The n_mix_—total number of items experimentally determined; n_add_—total number of items calculated for the additive two-drug mixture; *p<0.05 vs. the respective IC_50add_ value (Student-s t-test with Welch’s correction).

**Fig. 6:**
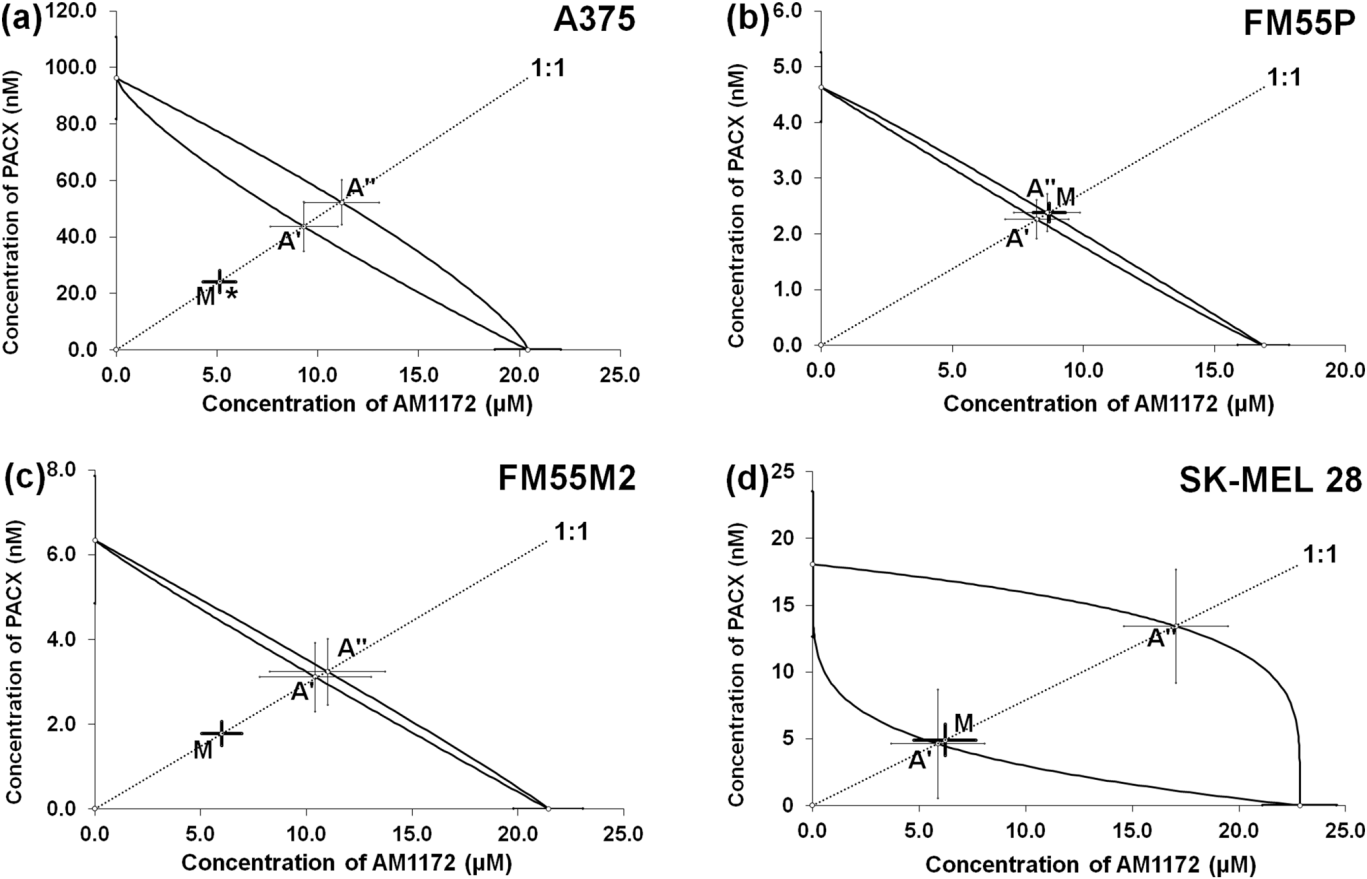
Isobolograms showing interactions between AM1172 and paclitaxel (PACX) concerning their anti-proliferative effects on A375 (**a**), FM55P (**b**), FM55M2 (**c**), and SK-MEL 28 (**d**) malignant melanoma cell lines measured in vitro by the MTT assay. Points A’ and A” depict the theoretically calculated IC_50add_ values for both lower and upper isoboles of additivity, respectively. Point M represents the experimentally derived IC_50mix_ values for the total concentration of the mixture of AM1172 with PACX that produced a 50% anti-proliferative effect in malignant melanoma cell lines measured *in vitro* by the MTT assay. *p<0.05, vs. the respective IC_50add_ value. Statistical analysis was performed with Student’s t-test followed by Welch’s correction.

Similarly, the combinations of AM1172 and PACX at the fixed ratio of 1:1 showed additive interactions for SK-MEL 28, FM55P, and FM55M2 cell lines (Fig. 6B–D, Table 1) and synergistic interaction for A375 cell line (*p<0.05; Fig. 6A, Table 1).

**Fig. 7:**
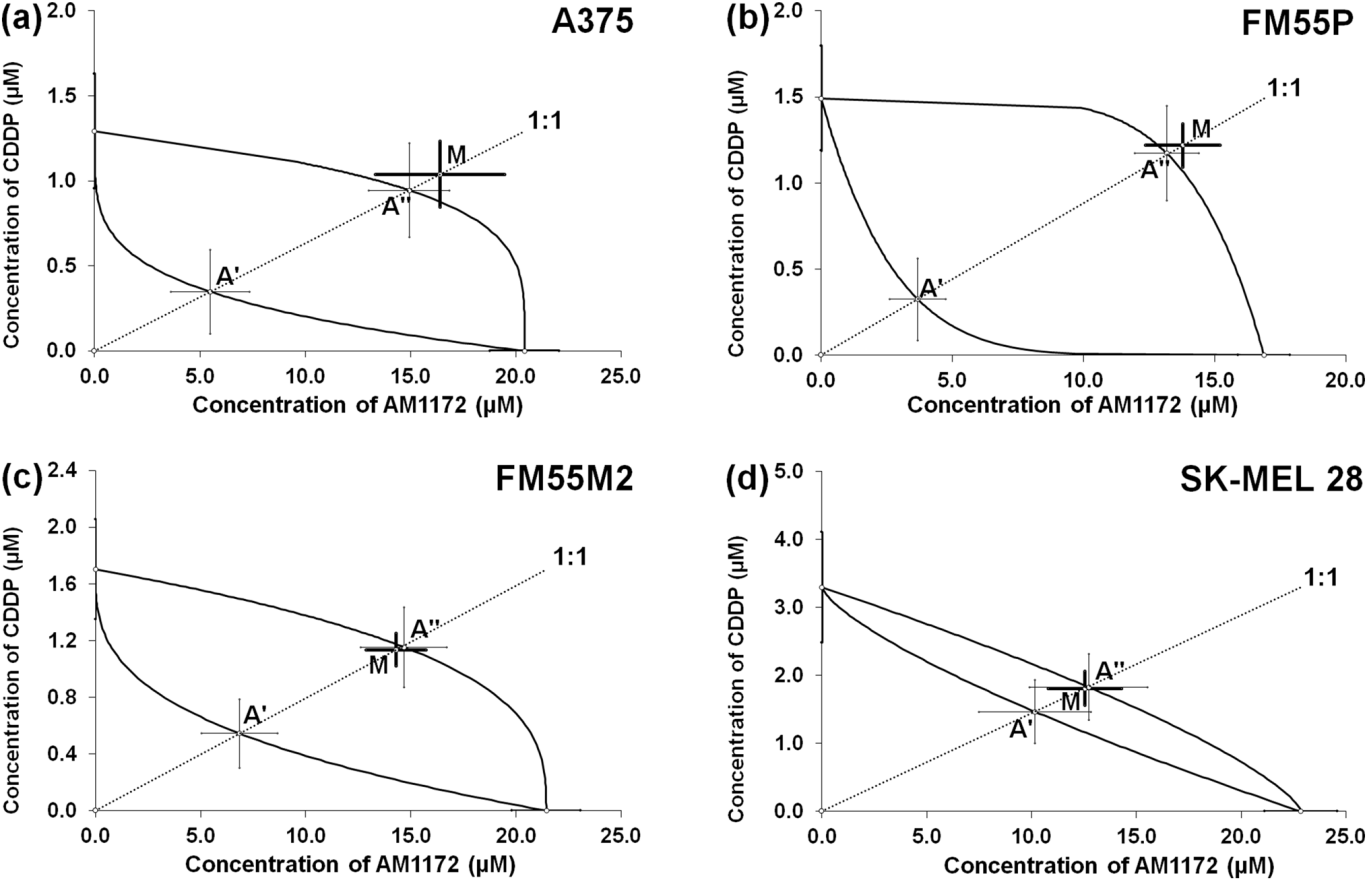
Isobolograms showing additive interactions between AM1172 and cisplatin (CDDP) with respect to their anti-proliferative effects on A375 (**a**), FM55P (**b**), FM55M2 (**c**), and SK-MEL 28 (**d**) malignant melanoma cell lines measured *in vitro* by the MTT assay. Points A’ and A” illustrate the theoretically additive IC_50add_ values for both lower and upper isoboles of additivity, respectively. Point M illustrates the experimental IC_50mix_ values for the total concentration of the mixture of AM1172 with CDDP that produced a 50% anti-proliferative effect in malignant melanoma cell lines measured *in vitro* by the MTT assay. Statistical analysis was performed with Student’s t- test followed by Welch’s correction.

The combination of AM1172 and CDDP at the fixed ratio of 1:1 showed additivity in all the studied melanoma cell lines (A375, FM55P, SK-MEL 28 and FM55M2) with a slight tendency towards antagonistic interactions in each (Fig. 7A–D, Table 1).

**Fig. 8:**
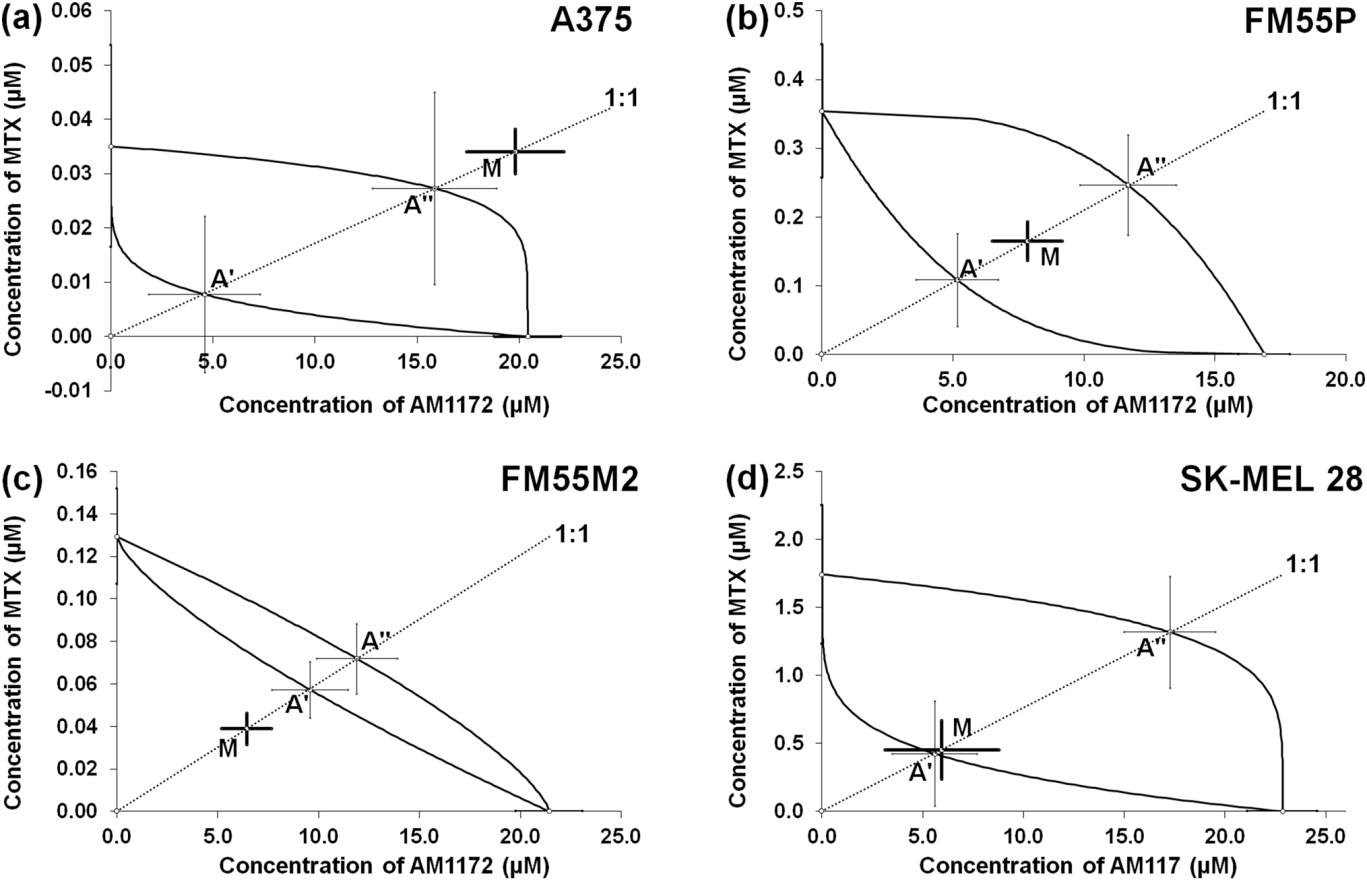
Isobolograms showing additive interactions between AM1172 and mitoxantrone (MTX) with respect to their anti-proliferative effects on A375 (**a**), FM55P (**b**), FM55M2 (**c**), and SK-MEL 28 (**d**) malignant melanoma cell lines measured *in vitro* by the MTT assay. Points A’ and A” illustrate the theoretically additive IC_50add_ values for both lower and upper isoboles of additivity, respectively. Point M illustrates the experimental IC_50mix_ values for the total concentration of the mixture of AM1172 with MTX that produced a 50% anti-proliferative effect in malignant melanoma cell lines measured *in vitro* by the MTT assay. Statistical analysis was performed with Student’s t- test followed by Welch’s correction.

The interactions of AM1172 and MTX at the fixed ratio of 1:1 were additive in each of the tested melanoma cell lines (A375, FM55P, SK-MEL 28, and FM55M2) (Fig. 8A–D, Table 1).

## Discussion

**Fig. 9:**
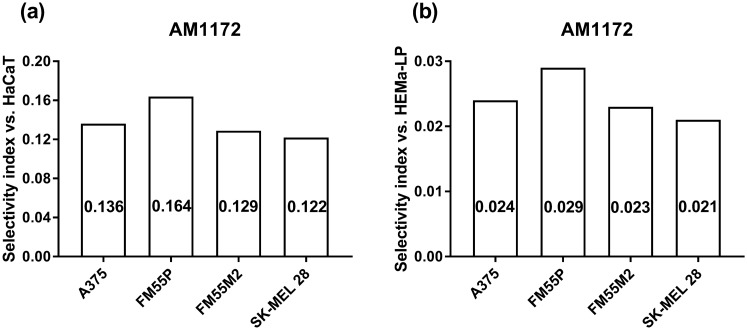
Comparison of selectivity index for AM1172 in the MTT assay. The selectivity index was calculated using the formula: *SI = (IC*_*50*_* for normal cell line)/(IC*_*50*_* for respective melanoma cell line*).

Results clearly indicate that AM1172, in a concentration-dependent manner, reduced cell viability in all of the tested malignant melanoma cell lines (A375, SK-MEL 28, FM55P, and FM55M2) in the MTT assay. Unfortunately, the concentrations of AM1172 that reduced cell viability in melanoma cells (1 – 15 µg/mL) were high enough and considerably reduced cell viability of normal human melanocytes (HEMa-LP) and human keratinocytes (HaCaT) in the MTT assay. In such a situation, the inhibition of AEA hydrolysis along with a consecutive increase in AEA concentrations and/or prolonged action of AEA on CB1 and CB2 receptors in response to AM1172-mediated effect (i.e., inhibition of cellular uptake of AEA) produced not only the antiproliferative effects in various human melanoma cell lines but also on normal human cells, suggesting that AM1172 cannot be used alone as a potential chemotherapeutic drug. To experimentally assess the cytotoxic effects of AM1172 on normal human cells and to determine the safety profile of AM1172, the selectivity index, as a ratio of the IC_50_ for normal cell lines (HaCaT and HEMa-LP) and IC_50_ for the respective melanoma cell lines (A375, SK-MEL 28, FM55P and FM55M2), was calculated. Generally, the tested compounds with selectivity indices higher than 1 indicate drugs with efficacy against tumor cells greater than the toxicity against normal cells [70]. In this study, the selectivity index for AM1172 was lower than 0.3 concerning keratinocytes (HaCaT cell line), and lower than 0.03 concerning melanocytes (HEMa-LP cell line) (Fig. 9). In contrast, it has been reported that the tested melanoma cell lines (A375, SK-MEL 28, FM55P, and FM55M2) were more susceptible than normal human keratinocytes and melanocytes on the antiproliferative effects of other cannabinoid ligands, i.e., arvanil, olvanil and CBD [22, 26].

Since AM1172 produced more expressed toxicity on normal human keratinocytes and melanocytes (non-tumor cells) than on malignant melanoma cells, this compound cannot be used separately as a treatment option in melanoma patients. On the other hand, it is difficult to unequivocally ascertain, which of the mechanisms of AM1172 is/are responsible for such toxicity on normal cells. Comparing the IC_50_ value of AM1172 from the SK-MEL 28 cell line (22.85 µM) with that determined from normal human HEMa-LP cells (0.49 µM), it was evident that the IC_50_ value for SK-MEL 28 was 46-times higher than that for HEMa-LP. In other words, to obtain the same 50% inhibitory effect on cell proliferation in the MTT assay (IC_50_), a 46-time higher concentration of AM1172 should be used in SK-MEL 28 than in HEMa-LP cell lines. In this study, it was serendipitously found that AM1172 was more toxic on normal human cells than on malignant melanoma cells. AM1172-evoked elevation in AEA concentrations in cellular microenvironment was deadly for normal cells, whereas the malignant melanoma cells developed some specific mechanisms making their cells resistant to the increased content of endocannabinoids. It is highly likely that any (even subtle) increase in endocannabinoid content in normal cells and/or overstimulation of the respective receptors by endocannabinoids resulted in a high rate of normal cell death. On the other hand, AM1172 by itself can evoke toxicity via unknown as yet mechanism(s). Nevertheless, all the above-mentioned hypothetical speculations on cytotoxicity in normal cells need detailed experimental verification in further biochemical and molecular studies. On the other hand, the main question on why the tested malignant melanoma cells were resistant to AM1172-induced cytotoxicity remains to be elucidated.

In the second part of this study, the anti-proliferative effects of the combinations of AM1172 with 4 commonly used chemotherapeutics (MTX, CDDP, DOCX, and PACX) in the MTT assay in 4 melanoma cell lines were determined. With isobolographic analysis of interaction, it was found that all the combinations of AM1172 with 4 tested chemotherapeutics exerted additive interactions in terms of reduction of cell viability in all of the studied melanoma cell lines, except for a combination of AM1172 with PACX in the A375 cell line, which occurred synergistic in the MTT assay. In other words, this unique combination offered a greater reduction in cell viability than was expected if considering the sum of anti-viability effects produced by AM1172 and PACX in the two-drug mixture on A375 cells. It is worth noting that concentrations of AM1172 used in the two-drug mixtures when combined with 4 commonly used chemotherapeutics were lower than those for AM1172 when used alone. It seems that combinations of chemotherapeutics with AM1172, which by itself elevates AEA content by inhibition of AEA cellular uptake and prolongs the action of AEA on CB1 and CB2 receptors, may become an alternative treatment option for melanoma patients. However, more advanced in vivo studies are required to confirm these experimental in vitro findings.

In the present study, the most potent was the combination of AM1172 with PACX, offering the synergistic interaction in the A375 cell line (Table 1, Fig. 6). Considering molecular mechanisms of the anti-viability action of AM1172 in combination with PACX in the A375 cell line, it should be stated that the synergistic cooperation of AM1172-mediated increase in AEA concentrations and prolongation of its action on the respective receptors inhibits the cell proliferation. On the other hand, PACX can stabilize microtubules and arrest mitosis, affecting the tumor microenvironment as well [71–73]. All these mechanisms contribute to the antiproliferative effects in melanoma cells. Briefly, synergistic collaboration of two drugs (PACX and AM1172) in terms of inhibition of melanoma proliferation indicates that both drugs independently inhibit melanoma cells, in contrast to the combination of other drugs with AM1172 for which additive interactions occurred. Although DOCX and PACX have similar molecular mechanisms of action, the combination of AM1172 with DOCX in the A375 cell line exerted additive interaction with a tendency towards synergy, suggesting that the mechanisms of both drugs are complementary in terms of suppression of melanoma cell proliferation. On the other hand, the combinations of AM1172 with CDDP exerted additive interactions in the MTT test, which are in contrast to the other cannabinoids tested earlier (i.e., arvanil, olvanil, and CBD) for which the interactions were antagonistic in almost all the tested cell lines (Table 2). Such a diversity in types of interactions of CDDP with various cannabinoid ligands and modulators of endocannabinoid activity may suggest competitive mechanisms of the anti-melanoma activity, which inhibited cell proliferation producing finally either additive or antagonistic interactions.

**Table 2:** Characteristics of interactions between selected cannabinoid ligands and MTX or CDDP in various malignant melanoma cell lines.

Drug combination	A375	FM55P	FM55M2	SK-MEL 28	Reference
CBD + MTX	A	A	A	A	[22]
CBD + CDDP	ANT	ANT	A	ANT	[22]
ARV + MTX	A	A	A	A	[26]
ARV + CDDP	ANT	ANT	ANT	ANT	[26]
OLV + MTX	A	A	A	A	[26]
OLV + CDDP	ANT	ANT	A	A	[26]
AM1172 + MTX	A	A	A	A	this study
AM1172 + CDDP	A	A	A	A	this study

*A* additivity, *ANT* antagonism, *ARV* arvanil, *CBD* cannabidiol, *OLV* olvanil

In this study, AM1172 combined with CDDP produced only additive interactions, which may be a result of different mechanisms of action, i.e., raising AEA levels by AM1172 [34–36], and CDDP-induced DNA lesions via interaction with purine bases on DNA, leading finally to cell apoptosis or necrosis by the two-drug mixture [74, 75]. The results for the combinations of AM1172 with MTX stand in line with the previous studies with CBD, arvanil, and olvanil, presenting the additive type of interactions in all the tested melanoma cell lines [22, 26].

Another fact deserves a short explanation when comparing the results obtained in the MTT assay for the FM55P and FM55M2 strains (Table 2). These two cell lines, despite their same origin, are primary and metastatic cell lines, respectively. Any differences observed between these two cell lines confirm the fact that both cell lines derived from different cell colonies, i.e., invasive metastatic and solid tumorous. Differences in the cell viability observed in the MTT assay for these two cell strains (FM55P and FM55M2) treated with two-drug mixtures confirmed indirectly some diversity in cell microstructure and/or receptor compositions, as well as different metabolic pathways activating apoptosis and cell death during exposition to the mixtures. At present, it is difficult to indicate the mechanism(s) responsible for the observed diversity in the anti-proliferative action.

The main limitations in this study are, among others: (1) toxic effects of AM1172 when used alone on healthy human skin cells that can restrict the potential use of the drug; (2) unknown as yet and/or poorly recognized signs of over-activation of the endocannabinoid system in vivo studies. Generally, the excessive activation of the endocannabinoid system may accelerate some physiological processes responsible for tumor inhibition and reduction of melanoma cell viability, but it may evoke some cannabinoid-specific adverse effects, being the result of excessive activation of CB1 and CB2 receptors. To overcome these limitations, the pre-treatment with AM1172 could alter cell hemostasis and provide a more pronounced effect on chemotherapy with DOCX, PACX, CDDP, or MTX conducted later. AM1172 through modulation of the endocannabinoid system in the cellular microenvironment could make malignant melanoma cells more sensitive to the chemotherapeutic treatment, bringing benefit during the treatment of malignant melanoma. Further experimental studies conducted on melanoma cell lines should shed more light on our knowledge of the involvement of the endocannabinoid system in cell proliferation in malignant melanoma.

## Conclusions

The isobolographic analysis of interactions of AM1172 with either DOCX, PACX, CDDP, or MTX provided evidence for the additive interactions in terms of reduction of cell proliferation, with the most favorable combination of AM1172 and PACX, producing synergy in the A375 cell line. Although the adjunctive therapy of AM1172 with PACX, DOCX, and CDDP can be considered as potentially favorable combinations in the treatment of malignant melanoma, the application of AM1172 can significantly diminish the viability and proliferation of the human healthy skin cells. AM1172-evoked changes in the endocannabinoid system, as a result of inhibition of AEA cellular uptake after a single administration of AM1172, may be harmful for normal healthy skin cells. In such case the utmost caution is advised to researchers and scientists before the application of AM1172 as the anti-proliferative drug.

### Supplementary Information

Below is the link to the electronic supplementary material.

Supplementary file1 Fig. 1 Concentration–effect lines for AM1172 and DOCX administered alone and in combination at the fixed ratio of 1:1, illustrating the anti-proliferative effects of the tested drugs in the malignant melanoma cell lines: A375 (a), FM55P (b), FM55M2 (c), and SK-MEL 28 (d) measured in vitro by the MTT assay. Test for parallelism confirmed that the experimentally determined concentration–effect lines for AM1172 and DOCX (administered alone) are mutually non-parallel to each other in A375, FM55P, FM55M2 and SK-MEL 28 cell lines (TIF 185 KB)

Supplementary file2 Fig. 2 Concentration–effect lines for AM1172 and PACX administered alone and in combination at the fixed ratio of 1:1, illustrating the anti-proliferative effects of the tested drugs in the malignant melanoma cell lines: A375 (a), FM55P (b), FM55M2 (c), and SK-MEL 28 (d) measured in vitro by the MTT assay. Test for parallelism confirmed that the experimentally determined concentration–effect lines for AM1172 and PACX (administered alone) are mutually non-parallel to each other in A375, FM55P, FM55M2, and SK-MEL 28 cell lines (TIF 182 KB)

Supplementary file3 Fig. 3 Concentration–effect lines for AM1172 and CDDP administered alone and in combination at the fixed ratio of 1:1, illustrating the anti-proliferative effects of the tested drugs in the malignant melanoma cell lines: A375 (a), FM55P (b), FM55M2 (c), and SK-MEL 28 (d) measured in vitro by the MTT assay. Test for parallelism confirmed that the experimentally determined concentration–effect lines for AM1172 and CDDP (administered alone) are mutually non-parallel to each other in A375, FM55P, FM55M2 and SK-MEL 28 cell lines (TIF 172 KB)

Supplementary file4 Fig. 4 Concentration–effect lines for AM1172 and CDDP administered alone and in combination at the fixed ratio of 1:1, illustrating the anti-proliferative effects of the tested drugs in the malignant melanoma cell lines: A375 (a), FM55P (b), FM55M2 (c), and SK-MEL 28 (d) measured in vitro by the MTT assay. Test for parallelism confirmed that the experimentally determined concentration–effect lines for AM1172 and CDDP (administered alone) are mutually non-parallel to each other in A375, FM55P, FM55M2 and SK-MEL 28 cell lines (TIF 182 KB)

## Data Availability

The datasets generated during and/or analyzed during the current study are available from the corresponding author upon reasonable request.

## Abbreviations

AEA: Anandamide
CB1: Cannabinoid receptor 1
CB2: Cannabinoid receptor 2
CBD: Cannabidiol
CDDP: Cisplatin
Δ^9^THC: Delta-^9^-tetrahydrocannabinol
DOCX: Docetaxel
FAAH: Fatty acid amide hydrolase
FABPs: Fatty acid binding proteins
GPR35: G-protein coupled receptor 35
GPR55: G-protein coupled receptor 55
MTX: Mitoxantrone
PACX: Paclitaxel
PEA: Palmitoylethanolamide
PPARα: Peroxisome proliferator-activated receptor alpha
PPARγ: Peroxisome proliferator-activated receptor gamma
ROS: Reactive oxygen species
TRPV1: Transient receptor potential vanilloid-1 channel
