# Supplementary figures and images for: AM1172 (a hydrolysis-resistant endocannabinoid analog that inhibits anandamide cellular uptake) reduces the viability of the various melanoma cells, but it exerts significant cytotoxic effects on healthy cells: an in vitro study based on isobolographic analysis
Source: Pharmacol Rep. 2023 Nov 29;76(1):154–70. doi: 10.1007/s43440-023-00557-2 (PMC10830817; doi:10.1007/s43440-023-00557-2)

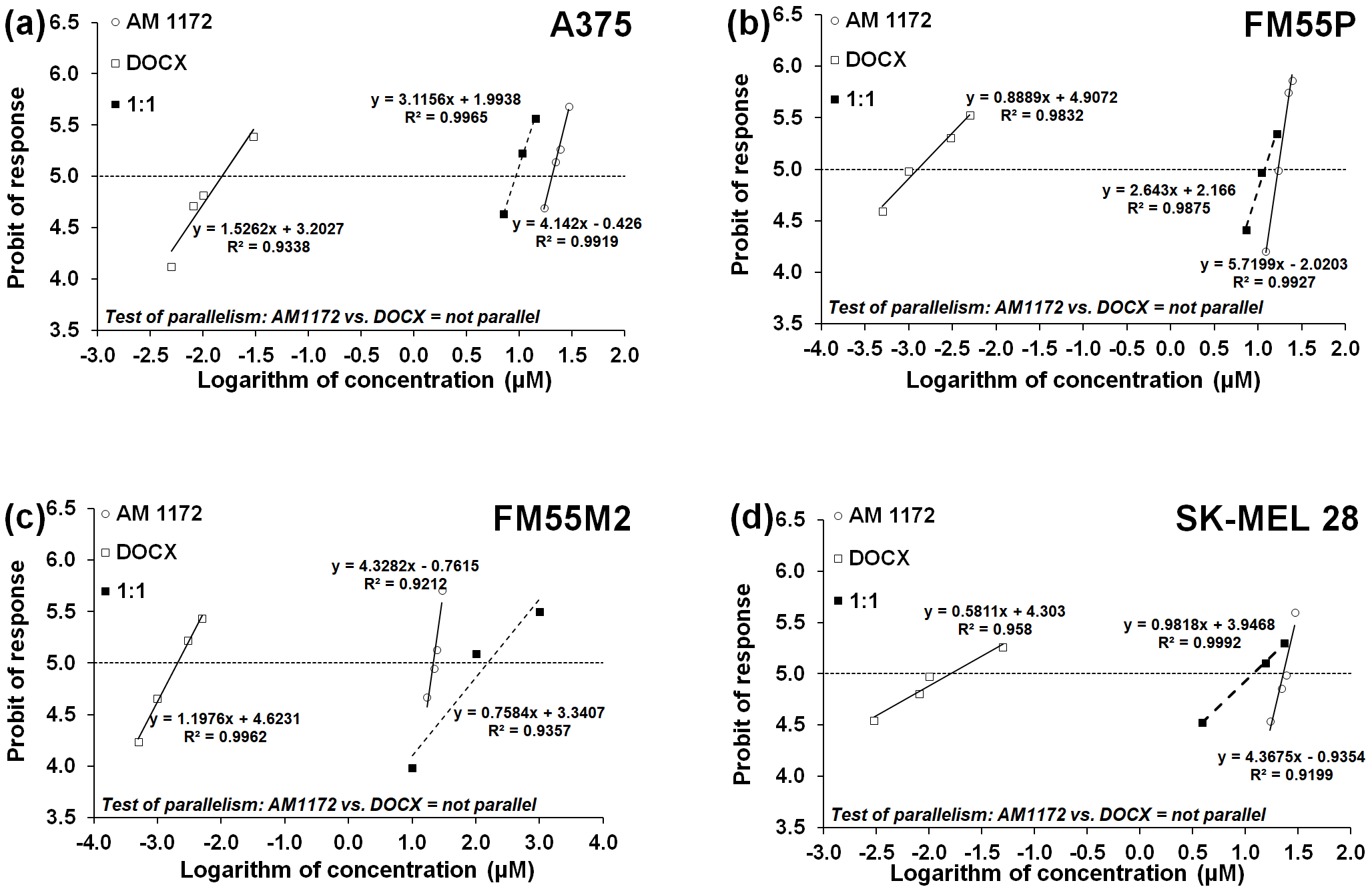

Supplement: Supplementary file 1 — Supplementary file1 Fig. 1 Concentration–effect lines for AM1172 and DOCX administered alone and in combination at the fixed ratio of 1:1, illustrating the anti-proliferative effects of the tested drugs in the malignant melanoma cell lines: A375 (a), FM55P (b), FM55M2 (c), and SK-MEL 28 (d) measured in vitro by the MTT assay. Test for parallelism confirmed that the experimentally determined concentration–effect lines for AM1172 and DOCX (administered alone) are mutually non-parallel to each other in A375, FM55P, FM55M2 and SK-MEL 28 cell lines (TIF 185 KB) [file 43440_2023_557_MOESM1_ESM.tif]

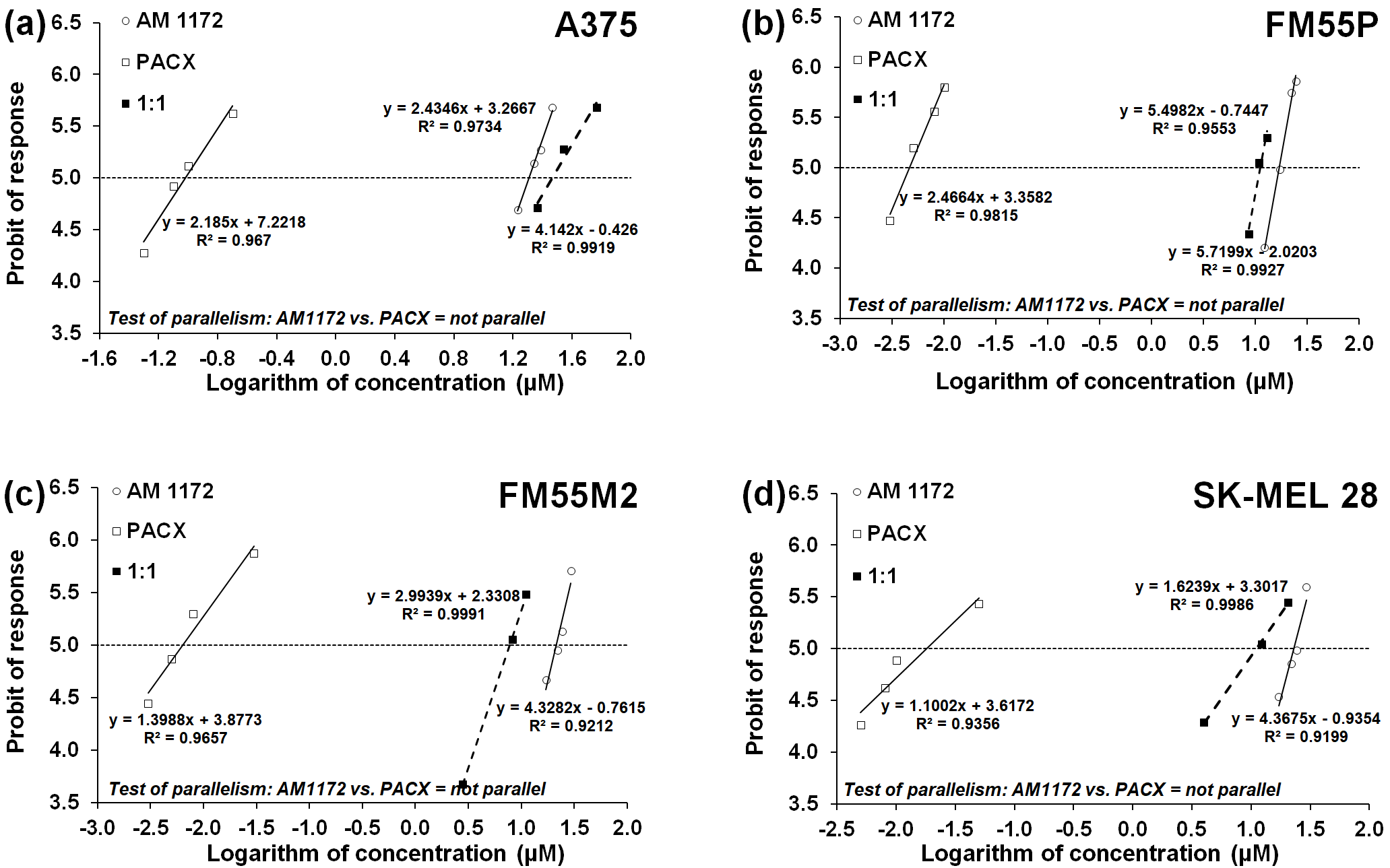

Supplement: Supplementary file 2 — Supplementary file2 Fig. 2 Concentration–effect lines for AM1172 and PACX administered alone and in combination at the fixed ratio of 1:1, illustrating the anti-proliferative effects of the tested drugs in the malignant melanoma cell lines: A375 (a), FM55P (b), FM55M2 (c), and SK-MEL 28 (d) measured in vitro by the MTT assay. Test for parallelism confirmed that the experimentally determined concentration–effect lines for AM1172 and PACX (administered alone) are mutually non-parallel to each other in A375, FM55P, FM55M2, and SK-MEL 28 cell lines (TIF 182 KB) [file 43440_2023_557_MOESM2_ESM.tif]

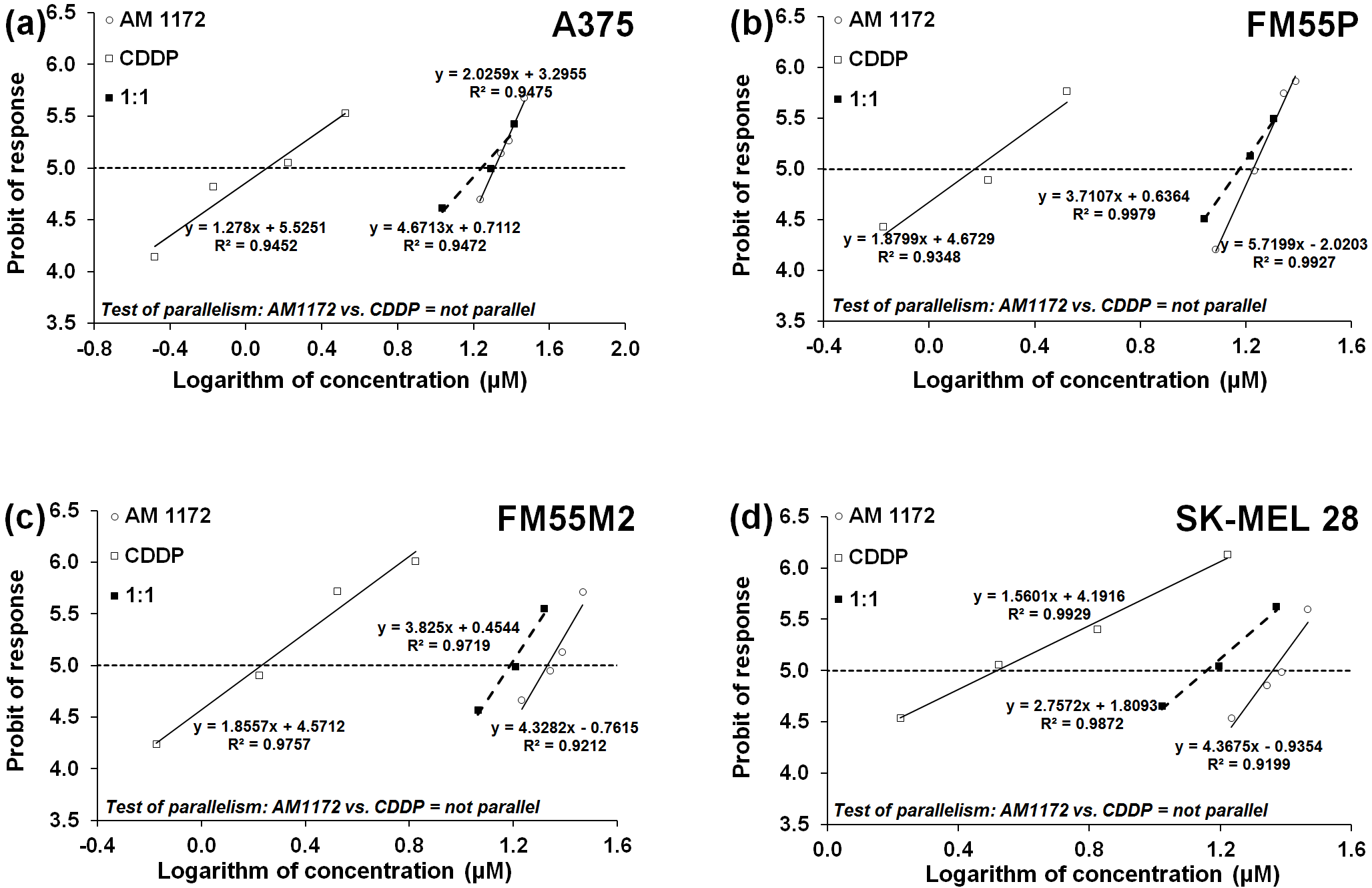

Supplement: Supplementary file 3 — Supplementary file3 Fig. 3 Concentration–effect lines for AM1172 and CDDP administered alone and in combination at the fixed ratio of 1:1, illustrating the anti-proliferative effects of the tested drugs in the malignant melanoma cell lines: A375 (a), FM55P (b), FM55M2 (c), and SK-MEL 28 (d) measured in vitro by the MTT assay. Test for parallelism confirmed that the experimentally determined concentration–effect lines for AM1172 and CDDP (administered alone) are mutually non-parallel to each other in A375, FM55P, FM55M2 and SK-MEL 28 cell lines (TIF 172 KB) [file 43440_2023_557_MOESM3_ESM.tif]

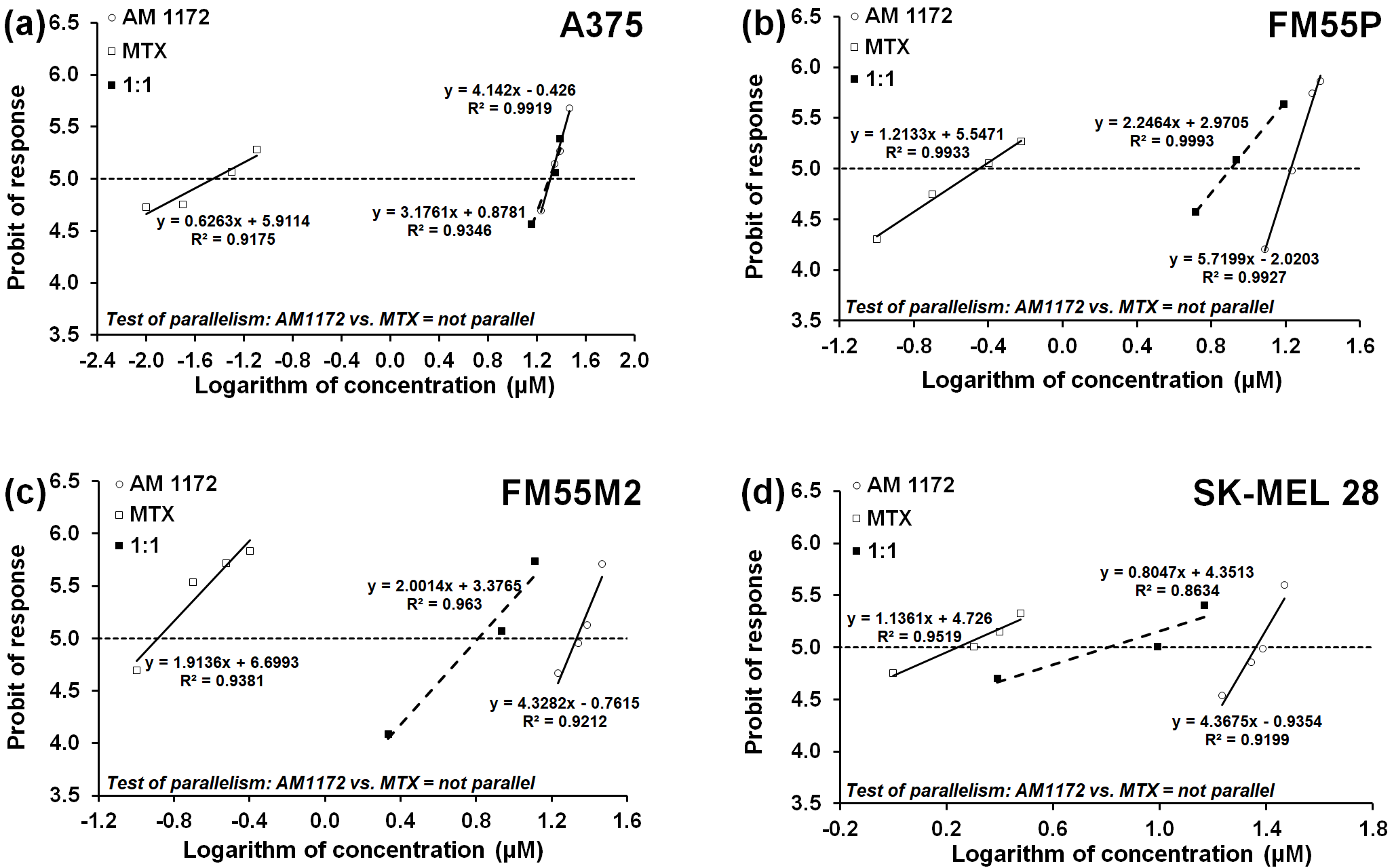

Supplement: Supplementary file 4 — Supplementary file4 Fig. 4 Concentration–effect lines for AM1172 and CDDP administered alone and in combination at the fixed ratio of 1:1, illustrating the anti-proliferative effects of the tested drugs in the malignant melanoma cell lines: A375 (a), FM55P (b), FM55M2 (c), and SK-MEL 28 (d) measured in vitro by the MTT assay. Test for parallelism confirmed that the experimentally determined concentration–effect lines for AM1172 and CDDP (administered alone) are mutually non-parallel to each other in A375, FM55P, FM55M2 and SK-MEL 28 cell lines (TIF 182 KB) [file 43440_2023_557_MOESM4_ESM.tif]
